# A Practical Treatment on Obstetrics

**Published:** 1887-06

**Authors:** 


A PRACTICAL TREATISE ON OBSTETRICS. *Vol. II, The Pathology of Pregnancy, and Vol. III, The Pathology of Labor, By* A. CHARPENTIER, M.D., *Paris, Illustrated with lithographic and wood engravings. These are Vols. II and III of "The Cyclopædia of Obstetrics and Gynæcology" —1887. New York:* WILLIAM WOOD & CO., *Chicago*: W. T. KEENER.

The first volume of this treatise has already been reviewed in this journal, and these volumes call for no special notice. The claim of the translator, that it is "the most complete treatise on obstetrics in any language," is fully sustained. The translator's notes continue to be a desirable addition to the work. E. W.

